# Deciding on the location for receiving parenteral antimicrobial therapy: development and preliminary testing of a patient decision aid

**DOI:** 10.1186/s12913-025-13434-w

**Published:** 2025-09-30

**Authors:** Marie Louise Thise Rasmussen, Dawn Stacey, Kirsten Lomborg, Hanne Konradsen

**Affiliations:** 1 Department of Emergency Medicine, Copenhagen University Hospital - Herlev and Gentofte, Herlev, 2730 Denmark; 2 School of Nursing, Faculty of Health Sciences, University of Ottawa, Ottawa, Canada; 3 Centre for Implementation Research, Ottawa Hospital Research Institute, Ottawa, Canada; 4 Department of Clinical Research, Copenhagen University Hospital - Steno Diabetes Center Copenhagen, Herlev, 2730 Denmark; 5 Department of Clinical Medicine, Faculty of Health and Medical Sciences, University of Copenhagen, Copenhagen N, 2200 Denmark; 6 Division of Nursing, Department of Neurobiology, Care Sciences and Society, Karolinska Institute, Stockholm, 171 77 Sweden; 7 Department of Geriatrics, Copenhagen University Hospital – Amager and Hvidovre, Hvidovre, 2650 Denmark

**Keywords:** Patient decision aid, Shared decision-making, Emergency department, Development, Healthcare delivery, Outpatient parenteral antimicrobial therapy, Denmark

## Abstract

**Background:**

Adult patients who need further treatment with parenteral antimicrobial therapy after a stay in the emergency department could be involved in the decision about the location of treatment. Although patients often get admitted to hospitals for parenteral antimicrobial therapy, they can also receive it in their home with different support. Therefore, this study aimed to develop and alpha test a patient decision aid to support patient involvement in this decision.

**Methods:**

A systematic development process was guided by the Ottawa Decision Support Framework. This process included developing a patient decision aid and conducting individual interviews with Danish patients and healthcare professionals to test the comprehensibility, acceptability, and usability of the aid. Directed content analysis guided the analysis.

**Results:**

An in-consult 2-page patient decision aid was developed to fit the busy environment of the emergency department. It included (a) a title specifying the decision; (b) information on the treatment, three options, advantages and disadvantages of the options; (c) a value clarification exercise; and (d) a question about preferred option. The patient decision aid met the qualifying criteria of the International Standards for Decision Aids. Participants’ feedback on the comprehensibility was positive, indicating explicit and clear options, and minor suggestions for editing. The healthcare professionals were reserved when asked about the acceptance and usefulness of the patient decision aid because the three options were not considered equal and were difficult to offer due to limited resources. The patients were also skeptical that their preferences could be considered in the decision-making, and they expressed uncertainty about whether treatment at home was as safe as hospitalization. The healthcare professionals recognized the importance of shared decision-making. However, the implementation of the decision aid would necessitate specific competencies, and identification of the best time to introduce it to patients.

**Conclusions:**

Using a systematic process, a patient decision aid was developed. Comprehensive findings revealed that the decision aid could be useful in supporting shared decision-making and in clarifying the available options. However, for the decision aid to be implemented, there needs to be a clear context and training of healthcare professionals in shared decision-making.

**Supplementary Information:**

The online version contains supplementary material available at 10.1186/s12913-025-13434-w.

## Background

Many patients prefer in-home care instead of hospital care, based on factors such as feeling comfortable, safe, being with relatives, and being away from a crowded environment [1]. Given current pressures on the healthcare costs, there are increased attempts to move patients out of hospitals faster by providing care at home or in community health clinics [2, 3]. Patients who need parenteral antimicrobial therapy (PAT) have different needs and preferences [4]. Some require bed rest while others can go about their normal lives without insurmountable barriers. Some live alone while others live together with family or friends who can assist them as needed. Some might lack self-assurance, while others feel comfortable with self-surveillance and self-care. Therefore, attention should be paid to both patient safety and individual circumstances, needs, and preferences when deciding upon the best location for treatment [4, 5].

Admission to a specialized ward can be avoided or the length of stay can be reduced if (a) healthcare professionals (HCPs) evaluate the patient to be in a clinical state that is considered safe for the patient to receive treatment at home, and (b) the choice of treatment is possible to administer in the patient’s home. The outpatient parenteral antimicrobial therapy healthcare service was introduced to provide treatment outside of the hospital setting in the USA in the mid-1970s [6]. The initial outpatient parenteral antimicrobial therapy model referred to a visiting nurse model, where primary care nurses infuse parenteral antimicrobials in patients’ homes. Over the years, many other models have been developed, including the self-administration model with periodic (e.g., once-a-day) support either by a visiting nurse or the patient attending an outpatient clinic [7]. These models aim to allow for the safe administration of parenteral antimicrobials outside the hospital and have been accepted in Canada, Australia, New Zealand, the United Kingdom, Italy, and Denmark [8, 9]. However, standard practice in Denmark is currently to be admitted to the hospital for PAT. Internationally, it has been reported that PAT has not yet been fully implemented, and it has been suggested that health systems across sectors should collaborate better and follow a more rigorous workflow [8]. Whether patients receiving PAT prefer to be treated at home rather than in a hospital has not yet been explored [9].

Given the complexity of the decision, shared decision-making (SDM) between patients and HCPs can be used to achieve person-centered care. SDM combines patients’ values for features of options with professional expertise and scientific evidence [10]. Patient decision aids (PtDAs) are tools that can facilitate SDM [11] and, in some cases, have positively affected clinical outcomes [10–12]. PtDAs can make explicit the decision to be made, provide information on options (including advantages and disadvantages), and play an essential role in assessing the values for features and outcomes of options [13]. In an emergency department (ED) where decisions about the patient’s further care trajectory are planned, a PtDA can summarize the advantages and disadvantages of available options to help patients choose the most appropriate care plan with their HCP.

Rigorously evaluated PtDAs have been used in the ED for adults presenting with chest pain [14] and children presenting with head trauma [15]. Compared to usual care, both PtDAs increased patient and parent knowledge and decreased decisional conflict [14, 15]. Furthermore, patients presenting with chest pain were more engaged in decision-making [14]. A review of the international inventory of PtDAs revealed none focused on the location for PAT [11]. Among the documented PtDAs in a Danish context, none is developed for use with patients in the ED [16]. A Danish PtDA with information about the different options on location for receiving PAT has been developed for patients in an infectious disease department [17], the PtDA was however, not developed for patients meeting acute illness, and no evaluation of its usefulness has been published. To the best of our knowledge, no PtDA has been developed to be used in the ED to explicitly invite to a dialogue between patients and HCPs about patients’ preferences for safety and level of self-management, when choosing the location for PAT treatment.

## Aim

This study aimed to develop and test a PtDA to support adults in the ED facing the decision about the location for receiving PAT.

### Methods

#### Study design

We used a systematic development process that was guided by the Ottawa Decision Support Framework [18]. For the preliminary user testing, we followed the alpha test described by the International Patient Decision Aid Standards (IPDAS) development team [19]. The process involved: (a) completing the online patient decision aid training program [18]; (b) using the tested Ottawa template to create the PtDA [18]; and (c) conducting alpha testing with potential users to evaluate content, usefulness, and acceptance of the PtDA [19].

This article is structured using the reporting guidelines of the “Standards for Reporting Qualitative Research” [20] and “Standards for Universal reporting of patient Decision Aid Evaluation” [21]. The Capital Regional Committee on Health Research Ethics reported that no formal ethical approval was required (no. 20068796).

### Theoretical frameworks

The Ottawa Framework is a well-evaluated approach for developing a PtDA to support people making health decisions [22]. According to this framework, decisional outcomes can be improved by designing decision support interventions, such as PtDAs, to address patients’ or surrogates’ decisional needs. Randomized controlled trials of decision aids – including decision aids based on the Ottawa Framework – have shown that patients or surrogates who use decision aids have improved knowledge about the decision, decreased uncertainty, feel clearer about their values, and participate more actively in the decision-making process [22, 23].

PtDAs developed following the Ottawa Framework also fulfil the International PtDA Standards for decision aids. The International Standards provide a quality framework with a set of criteria based on theoretical and empirical evidence [24, 25]. More specifically, there are three categories of criteria: (1) qualifying criteria, (2) certification criteria to minimize biased decisions, and (3) quality criteria [26]. The qualifying criteria include six items that must be met for a tool to be considered a PtDA. The PtDA must (1) ´Describe health condition or problem for which index decision is required´, (2) ´Explicitly state decision under consideration (index decision)´, (3) ´Describe the options available for the index decision´, (4) ´Describe the positive features of each option´, (5) ´Describe the negative features of each option´, and (6) ´Describe the features of options to help patients imagine the physical, social and/or psychological effects´ [26, p. 463]]. These six items are used on the checklist for including a PtDA on the International A to Z inventory of publicly available PtDAs [27].

### Drafting the PtDA

The methods for drafting the PtDA followed the Ottawa Framework and used the well-established Ottawa template. Evidence on PAT options (including benefits and risks) was informed by a recently published Health Technology Assessment report [9]. Specific decisional needs identified in this report included getting the opportunity to be treated with PAT at home, having access to clear information about the treatment process, and knowing who to contact when help is needed [9]. These decisional needs were addressed in the PtDA.

The first author drafted the first version of the PtDA while completing the six modules in the online Ottawa Framework PtDA training program [18]. Within the training program, the IPDAS synthesis papers are embedded to provide more details on how to balance information and how to clarify values [28–32]. The second author reviewed and supervised the different stages of PtDA development. To ensure the PtDA was relevant to the target users in the ED setting, workflows were considered when selecting the PtDA format, layout, and size. As part of the development process, the draft PtDA was appraised to ensure it met the six qualifying criteria of the International Standards. This version of the PtDA was presented to the leader of the ED and a specialized discharge team in the ED and no changes were made to the PtDA.

### Setting and participants for alpha testing

The alpha test was conducted in the ED of a medium-sized university hospital in Denmark. In 2022, 3982 patients had a medical condition requiring treatment with PAT. After initiating PAT in the ED, 3978 patients were hospitalized for further PAT and then, after a few days, discharged home on oral antibiotics. Four patients were not hospitalized and were discharged directly from the ED with further PAT.

The alpha testing was performed to explore the PtDA’s acceptability (i.e., overall suitability for decision-making), usability (i.e., applicability in clinical practice), and comprehensibility (i.e., the understandability of its content). We conducted interviews for alpha testing with both patients and HCPs, as recommended by the IPDAS development team [19]. Eligible patients for alpha testing the PtDA were adults (≥ 18 years) currently hospitalized for PAT, able to review materials and respond to questions in Danish. Patients were excluded if they were too sick to participate or had limited cognitive abilities. Eligible HCPs worked in the ED. Both groups were purposefully sampled across age and sex and, in the case of HCPs, representatives of both nurses and physicians with different years of experience working in ED.

### Data collection

After verifying eligibility and obtaining written informed consent, we collected demographic data on the participants’ age, sex, level of education, occupation, and work experience. The participants participated voluntarily without compensation for their time, and the HCPs participated during their working hours. We interviewed the participants using an interview guide, which included targeted questions about comprehensibility, usability, and acceptability of the current workflow in the ED. The alpha test recommendations made by the IPDAS development group informed the interview guide [25, 33].

Before the interview, each participant was encouraged to read the paper-based PtDA, complete it, and write comments. The interviewer used a “think aloud” approach to get the participants to verbalize their immediate thoughts while reading the PtDA. Then they were asked questions about clarity of content, format, usability, and implementation in current clinical practice, which were based on the validated Ottawa Framework “User manual – Acceptability” [34] (see interview guides). The interviews were audio-recorded and conducted in January and February 2023.

### Data analysis

The interviews were analyzed using directed content analysis [35] to consider the perspectives of the patients and HCPs. The analysis consisted of four steps. First, two authors (first and last author) carefully listened to the interviews to acquire an understanding of the content about the predetermined categories outlined for alpha testing: *acceptability*, *usability*, and *comprehensibility*. In the second step, all authors identified groups of codes that shared the same common characteristics belonging to each category. We coded the data with the patient’s perspective and the HCP’s perspective separately. The third step, the analysis of the category *comprehensibility*, ended on a descriptive level, while the analysis of the category’s *acceptability* and *usability* included an interpretation level with identified subcategories. Finally, all authors audited, discussed, and reached a consensus on the findings.

### Findings

#### The PtDA

The development team co-designed an in-consult 2-page PtDA to fit in the busy ED (the PtDA is attached). The final PtDA met al.l six qualifying criteria of the International standards [26].

The content of the paper-based PtDA included; (1) a title that identified the decision *“Would you prefer to receive intravenous (IV) antibiotics in the hospital or at home? A decision aid to discuss your options with the nurse”*; (2) an introduction that provided information about the PAT treatment; (3) the three options for PAT location; (4) health and social factors that may influence the choice; (5) information on the advantages and disadvantages of the different options with an explicit values clarification exercise; and (6) a question asking about their preferred option.

Three possible options to get PAT were identified: Option (1) Stay at the hospital; Option (2) Go home with support from primary acute care nurses; and Option (3) Go home with a pump and support from the ED. According to the Danish Health Technology Assessment, none of the options are considered better than the others [9]. To help patients understand the features and outcomes of the options, information was then provided on the advantages and disadvantages of each option. For the values clarification exercise, patients were asked to rate with stars on a scale of zero to five what mattered most about the advantages and disadvantages (zero was not important, and five was extremely important). This exercise was intended to facilitate a discussion between the patient and nurse on patient preferences to inform a SDM process. Finally, patients were asked about their overall preference for location when receiving PAT. Within the current workflow, the PtDA was designed to be used with the patient and nurse after a physician has concluded that the patient can be presented with different options for PAT.

### The alpha testing

#### Participants

The evaluation of the *acceptability*, *usability*, and *comprehensibility* of the PtDA was conducted with six patients receiving PAT in the hospital and 14 HCPs from the ED. The age range of the patients was between 31 and 82 years, equally distributed between both sexes and their education level varied from unskilled to higher education. The HCPs were eight nurses, one physiotherapist, and five physicians, of whom five were managers. Among the HCPs, the age range was between 28 and 60 years, six male and eight female. Their working experience in the ED ranged from six months to ten years and 2.5 to 38 years since graduation. The interviews lasted 10 to 40 min; the average length with patients was 14 min, and the average length with HCPs was 23 min. This variation in length was mainly due to the HCPs’ wanting to provide information about the procedures for discharging patients with intravenous antibiotics at home.

### The comprehensibility of the PtDA

The participants’ feedback on the *comprehensibility* of the PtDA was predominantly positive. The options were described as explicit.

*The options are well described. No information is missing* [to decide] (Patient 14), and *“Good written information. The patients would be prepared*,* and it* [the PtDA] *can push them in the direction that there are also other options* [than staying at the hospital]. (HCP 15)

The PtDA communicated information using plain language *“It is written in a language that is understandable* (Patient 8), and *“It is well formulated*,* and it speaks to the patients”* (HCP 7).

The level of detail for the information provided depended on the user. The patients found the information sufficient and clear *“The information is well laid out. Nicely clear and I do not miss any information”* (Patient 8). The HCPs worried about whether the patients would find it to be too much information and give up on trying to understand.

*It is very detailed and has long sections. To many* [patients] *who do not have a higher education*,* it could be difficult.* (HCP 15)

Suggestions for revising the PtDA included minor edits; adding health and social factors concerning reflections on the home environment, drug/alcohol use, and mental health, and implementing alternative ways of ranking the preferences.

### The acceptability of the PtDA

We identified two subcategories for acceptability. Participants described *Choosing must make sense* and *The options are not equal.*

**Choosing must make sense.** Both patient and HCP participants were challenged regarding the concept of SDM about the location for receiving PAT.

Patients expressed that even though they were invited to be involved in the decision by being given the PtDA, they were skeptical about whether their indicated preferences would be considered in the decision-making process. From the patients’ perspective, eliciting patient preferences would not make sense because the actual choice would depend on the available resources. Patients assumed that resources such as available beds in the hospital or staff among the primary acute care nurses would drive the choice of location.

*The stars* [referring to value clarification of preferences] *do not have any significance. It is the available resources that decide.* (Patient 10)

At the same time, patients did not always want to be presented with choices because they preferred not to be involved in the decision-making. *“I prefer to be free from making decisions”* (Patient 2).

The HCPs did not always find patient involvement in decisions necessary. From their view, they were able to make the right choice for the patient based on their professional education, and therefore, choosing together with the patient did not always make sense.

*It cannot always be up to the patient. We are the HCPs*,* and*,* in our care*,* we consider what is right for the individual patient.* (HCP 4)

The first option, which was to stay at the hospital, was not perceived as a real option to choose from both the patient’s and HCP’s perspectives. Being hospitalized would only be for a patient needing it according to illness severity. Otherwise, if the patient had mild symptoms, the patient had to be discharged. However, the second and third options, which were going home with different levels of support, were considered as actual options to choose from.

*If it is not strictly necessary to be hospitalized*,* it is a waste* [of bed] (Patient 1), and

*Option one* [stay at the hospital] *is a necessity as you must stay. It is not a choice. However*,* options two and three* [going home with treatment] *is like you* [referring to the patient] *can cope well at home and we* [the HCPs] *want to discharge you with intravenous antibiotics.* (HCP 13)

**The options are not equal.** The participants, both patients and HCPs, were doubting whether the options were equal because they were not convinced that home treatment was possible. The prerequisites for home treatment felt unclear. The patients expressed insecurity toward the risk of underestimating how sick they were when they considered the possibility of going home with treatment – *“I find it difficult to judge when you are well enough to go home with treatment”* (Patient 2). The HCPs expressed a need for guidelines to navigate safety and hygiene for home treatment.

*There is a need for criteria regarding treatment at home. Intravenous antibiotics are potent medicine. There is direct access* [referring to the intravenous line] *and the home is not a clean environment as the hospital.* (HCP 4)

Furthermore, the participants found monitoring and access to clinical staff important during treatment. The patients wanted to feel safe and not be left on their own, and therefore, they preferred to stay at the hospital for treatment - *“I will feel most safe in the hospital”* (Patient 14). The HCPs were concerned about discharging patients to treatment at their homes because then the HCPs would not be able to follow the patients’ conditions. Patients receiving PAT were considered to need close observation.

*PAT at home – we must remember that it is not an option like ‘hospital at home’. Someone will get the medicine ready for infusion*,* but no nurses or doctor at home follow the effect and the need for more blood samples.* (HCP 17)

### The usability of the PtDA

Exploring the usability of the PtDA, we identified three subcategories: *On paper*,* options are accessible*, *The HCPs require skills and time to use the PtDA*, and *Use depends on illness severity and care trajectory.* Our findings acknowledged that the PtDA was usable for presenting options but described that the applicability of the PtDA in clinical practice was difficult and required the right moment of introduction to the patient.

**On paper options are accessible.** From the patients’ and the HCPs’ perspectives, the PtDA elucidated all options to be discussed - *“It is nice to be presented with the different options”* (Patient 14) and *“A good tool to illustrate both to the patient and the HCP that there are options for treatment”* (HCP 7). However, not all options were perceived as manageable, and therefore, some indicated options as accessible on paper but not in clinical practice. The patients expressed worries about insecurity with being sent home with treatment in the primary healthcare sector and challenges being assisted in situations such as getting anti-nausea medicine.

*I feel uncertain* [referring to going home with treatment]. *The second option*,* where the nurse is coming to you*,* you do not have to manage it by yourself. But I have had a lot of nausea*,* and then I just had some anti-nausea medication here. Maybe they could take care of that too – I do not know.* (Patient 2)

The HCPs found it difficult with a tool presenting options which they experienced not being able to meet the requirements in clinical practice because of limited access to equipment and enough colleagues both in their department and in the primary healthcare sector.

*The PtDA presents options to choose from. But I cannot see how it should fit together when we neither have the equipment nor the manpower for that* [referring to patients going home with treatment]. (HCP 16)

**The HCPs require skills and time to use the PtDA.** Regarding the applicability of the PtDA in the clinical workflow, the HCPs had concerns about the length of time and their skills in SDM.

The HCPs expressed having limited time to discuss decisions because, even though they found the dialogue relevant, the patients would have questions that needed to be answered and that would take time – *“It will be good to have the dialogue*,* but we do not have time”* (HCP 21). Furthermore, *It is a troublesome workflow”* (HCP 6) of discharging the patient with treatment, and it was easier to hospitalize patients receiving PAT. At the same time, the HCPs experienced missing skills about how to perform SDM, which was required to be taught – *“A conversation about preferences is needed*,* but we must learn it* (HCP 15)

**Use depends on illness severity and care trajectory.** Finding the right moment to introduce the PtDA in the ED was not straightforward.

The concerns from the patients’ perspective were that many things happened when they arrived in the ED, and they needed *“The situation must have calmed down before I can decide”* (Patient 14). The HCPs expressed that the condition of the patient had to be considered before introducing the PtDA because the patient could be too ill to familiarize themselves with the PtDA - *“When the patient is in the acute phase of illness*,* they are afraid and feeling unsafe”* (HCP 21). At the same time, the care trajectory of the patient had to be defined to know whether the options could be presented to the patient – “*It is not possible right away in the ED”* (HCP 21). To be usable, the PtDA introduction needed to be at another time or place, which was not further defined by the participants.

## Discussion

We developed a PtDA to be used in an ED setting when patients are facing the decision about the location for receiving PAT. Our findings revealed positive feedback from patients and HCPs on the comprehensibility of presenting the options clearly and explicitly.

In the development process, we focused on making the PtDA as brief as possible to accommodate the high-speed environment of the ED. This corresponds to the knowledge that brief in-consultation paper-based tools are preferred because they are experienced by the HCPs to fit better with existing practices [36]. However, we found a difference in the evaluation of the needed level of information between the patients and HCPs. In our study, patients with a low level of education were represented and had no problems with what some HCPs expressed as a lot of information that could be difficult for this group of patients. This contradiction between what HCPs believe and what patients express has also been found due to other aspects of decision-making, such as it is too complicated for patients to know how to choose and patients prefer that the HCPs decide on their behalf [37], while patients desire to be involved [38]. Attention to explore the reasons for this disconnect is needed to avoid further misinterpretation.

Our findings showed that HCPs had reservations about the acceptance and usefulness of the PtDA to fit into clinical practice. The HCPs in our study reported that they would require skills and needed extra time to use the PtDA. In a study testing Chest Pain Choice decision aid in ED, HCPs had received training in SDM before being introduced to the decision aid [14]. Lack of knowledge about SDM and not having confidence in one’s own SDM skills may hinder the use of a PtDA [36]. Training may encourage HCPs to reflect on and adjust their practice when their SDM competencies have been developed [39], but no studies have yet identified specific outcomes of a training program [40]. Concurrently, patients and HCPs had assumptions about the healthcare system not being ready for alternative care options other than hospitalization when further treatment with PAT is needed. The HCPs had experienced challenges when trying to discharge patients with PAT at home. However, there is a difference in the perspective from which the different participants speak: the patients answer from their own experiences, while the HCPs speak from a workflow and a professional perspective, and the managers from an organizational perspective. With further testing other factors may emerge. Very few patients had been discharged directly from the ED with PAT at home, which shows that it is possible but not routinely considered. Although other studies reported that patients were worried about feeling left on their own at home [41], these concerns could be discussed in a SDM encounter when discussing patients’ values and preferences.

The patients and HCP participants questioned whether there was a genuine choice and questioned necessity of a PtDA. A recently published study showed that a PtDA cannot stand alone but needs a supporting change of culture, attitudes, and management if SDM is to become routine care [42]. Further exploration on this view and workflow for discharge of patients with PAT at home is required before conducting any further testing of the PtDA.

There are several strengths and limitations to consider. Details on setting, characteristics of the participants, data collection, and analysis have been provided to allow the reader to determine whether the findings are transferable to similar contexts. The findings are from various perspectives of patients (ages, sexes, and education levels), and HCPs (ages, sexes, working experience in the ED, and different professions). Levels of health literacy were not assessed in this study. Having purposefully sampled participants based on level of health literacy might have been valuable to ensure including the diverse population attending the ED. The PtDA was alpha tested in one Danish ED setting, which may affect the transferability of the findings. The decisional needs were informed by the Health Technology Assessment [9]; however, this knowledge was collected from nine international studies, whereof none were Danish. The IPDAS recommends a systematic involvement of patients, HCPs, and primary care during the process of developing a PtDA [25]. Including these perspectives in the early stage of the development process of the PtDA could have contributed to the usefulness of the PtDA. For example, if, in practice, the option of hospitalization was not possible, the PtDA could have been adjusted to only include two options of in-home treatment. It can be considered a limitation that systematically collected knowledge from these groups through the development stages was not collected and included. Further testing of the PtDA will include a Beta testing, which will potentially reveal areas for improvement besides those identified in the Alfa test.

## Conclusion

We developed an evidence-based PtDA to facilitate SDM regarding the location for receiving PAT after an ED visit. Although comprehensive findings revealed that the PtDA could be useful in clarifying the available options and supporting SDM, further revisions may be needed to increase the acceptability and usability of the PtDA. Training of HCPs to address knowledge and skills and the timing of the introduction of the PtDA must be explored further to determine the right place and moment.

## Supplementary Information

Below is the link to the electronic supplementary material.

Supplementary Material 1

Supplementary Material 2

Supplementary Material 3

Supplementary Material 4

## Data Availability

The dataset(s) for this article are available upon reasonable request from the corresponding author.

## Abbreviations

ED: Emergency department
HCP: Healthcare professional
PAT: Parenteral antimicrobial therapy
PtDA: Patient decision aid
SDM: Shared decision making
