# Supplementary material for: Deciding on the location for receiving parenteral antimicrobial therapy: development and preliminary testing of a patient decision aid
Source: BMC Health Serv Res. 2025 Sep 30;25:1240. doi: 10.1186/s12913-025-13434-w (PMC12482543; doi:10.1186/s12913-025-13434-w)
Supplement: Supplementary file 1 — Supplementary Material 1 [file 12913_2025_13434_MOESM1_ESM.docx]

| **DA from the start to the end – the different parts** | In general | Acceptability | Your immediate thoughts about the tool?  What would you prefer – paper format or digital? And why?  How do you think the tool will affect your work? (a help?, save time?)  Would you use the tool? Why?/Why not? |
| --- | --- | --- | --- |
|  |  | Usability | What will be important for you when you use this tool?  Will you have to change your workflow? |
|  | What is it about | Usability | Is there information you think the patient lacks in order to make a qualified choice? |
|  | Options | Acceptability | When you read the choices, what do you think? |
|  | Health- and social factors | Usability | Could there be other health- and social factors that would influence the fact that it would be unsafe to send the patient home with iv treatment? |
|  | What matters most | Acceptability | Advantages and disadvantages – what will it mean for you to have a talk with the patient about what needs they have? |
|  |  | Usability | How do you understand the patient´s scores and how would you use them? |
| **Ending** |  |  | Is there anything you would like to add? |
