# Supplementary material for: Deciding on the location for receiving parenteral antimicrobial therapy: development and preliminary testing of a patient decision aid
Source: BMC Health Serv Res. 2025 Sep 30;25:1240. doi: 10.1186/s12913-025-13434-w (PMC12482543; doi:10.1186/s12913-025-13434-w)
Supplement: Supplementary file 2 — Supplementary Material 2 [file 12913_2025_13434_MOESM2_ESM.docx]

| **DA from the start to the end – the different parts** | In general | Comprehensibility | Is the tool written in a language that can be understood?  How is the design (layout and pictures)?  Is the font size good?  (more Dots?) |
| --- | --- | --- | --- |
|  |  | Usability | If you were given the tool and asked to fill it in – what would it be like? (easy/difficult?) |
|  | What is it about | Comprehensibility | Can you use the information?  Is there information you are missing? |
|  | Options | Comprehensibility | When you read the options, what do you think?  What do you understand by having choices?  Would it make sense for you to have these options? |
|  | Health- and social factors | Comprehensibility | Would these factors influence your choice? |
|  |  | Usability | Are there other factors than those mentioned in the tool that will influence your choice? |
|  | What matters most | Comprehensibility | Advantages and disadvantages – do they make sense?  Are there any advantages to the various options that you think are missing?  Are there any disadvantages to the various options that you think are missing? |
|  |  | Usability | How do you understand that you have to fill in this part?  Have you tried putting the stars – what is 0 not important and what is 5 very important?  And what does it look like/or would it look like for you? |
| **Ending** |  |  | Is there anything you would like to add? |
