# Supplementary material for: Deciding on the location for receiving parenteral antimicrobial therapy: development and preliminary testing of a patient decision aid
Source: BMC Health Serv Res. 2025 Sep 30;25:1240. doi: 10.1186/s12913-025-13434-w (PMC12482543; doi:10.1186/s12913-025-13434-w)
Supplement: Supplementary file 3 — Supplementary Material 3 [file 12913_2025_13434_MOESM3_ESM.pdf]

Would you prefer to receive intravenous (IV) antibiotics in the hospital or at home?

A decision aid to discuss your options with the nurse

This decision aid is for you if:

- You have an infection and need to take IV antibiotics
- You are offered a choice to have them in the hospital or at home

Infections requiring IV antibiotics

Antibiotics are medicines given to kill bacteria. IV antibiotics are stronger than when taken as a pill by mouth.

In the emergency department, your blood will be tested to identify the type of infection (e.g., blood cultures). While you wait for these results which can take 3 days, you will be started on a general antibiotic known to work with many different infections. The antibiotics are in a plastic bag attached to an IV line that the nurse inserts into a vein in your hand or arm.

Sometimes the blood culture results require a new antibiotic. The number of days you will have IV antibiotics will depend on the type of infection. The professionals of the emergency department will give you more details about the type of infection you have and the usual length of time for treatment with antibiotics.

What are your options?

There are three options for receiving IV antibiotics. No research studies have shown that one option is better than the other.

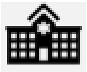

option 1

Stay at the **hospital** where the nurse will give you antibiotics **three times a day**. The nurse will also check how you are doing.

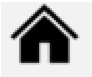

option 2

Be at your **home** with the **acute team** giving you antibiotics **three times a day**. Every infusion takes around 40 to 60 minutes. You bring the antibiotics with you when you leave the hospital. The acute team will check on how you are doing and are either staying during the infusion or leaving. You will have a phone number for the acute team if you have questions or need assistance.

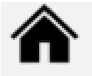

option 3

Be at your **home** with a **pump** that gives you the antibiotics **continuously. Every 24 hours** you must go into the emergency department where you will be checked, and your pump will be filled again. From home, you can telephone the emergency department if you have questions.

What (other) health and social factors may affect your choice?

Check ☑ any that apply to you and discuss with your HCP.

☐ Limited vision	☐ Very severe symptoms of infection
☐ Poor hearing	☐ No one to help you at home
☐ Diabetes	☐ Need help with managing my care
☐ Rheumatic diseases	☐ Take care of someone else
☐ Previous side effects of antibiotics	☐ No or little access to transport

☐ **Other**______________________________

☐ **None** of these apply to me

What matters most to you?

	**Reasons to Choose this Option** Advantages	How much it matters to you: 0★ not at all 5★ a great deal	**Reasons to Avoid this Option** Disadvantages	How much it matters to you: 0★ not at all 5★ a great deal
Option1 Stay at the hospital	Health professionals take care of me		Less privacy	
	Access to professionals 24 hours a day		Less control over my daily routines	
	Be able to recover outside my own home		Not being able to go to work	
	Less risk of being a burden on my family/friend(s)		Limited access to family/friend(s)	
Option 2 Be at home with support from the acute team	Health professionals giving me antibiotics		Professionals come to my home	
	Daily visits from health professionals who check how I am doing		Must be available at certain times at my home for scheduled visits of health professionals	
	Closer to family/friend(s)		May be a burden on my family/friend(s)	
	Sleep in my own bed		Feel like my home is a medical clinic	
Option 3 Be at home with a pump	Privacy		Feel more alone with managing my treatments	
	Feel in control of my time and life		Feel like my home is a medical clinic	
	Closer to family/friend(s)		May be a burden on my family/friend(s)	
	Able to go into work		Worry about not being able to manage my IV line and pump	
	Sleep in my own bed		Travel every day to the emergency department	

Now, think about which option has the reasons that are most important to you…

Which option do you prefer?
Check ☑ one.

☐ Option 1: Stay at the hospital to get your treatment
☐ Option 2: Be at home with support from the acute team
☐ Option 3: Be at home with a pump and with support from the emergency department

☐ Before making this decision, I will discuss it with ___________________________
☐ Unsure, because_____________________________________

This information is not intended to replace the advice of a health care provider.
Last reviewed: Fall 2022.
Content editors: MLTR
The development has received funding from the University of Copenhagen, ´Frimodt-Heineke Fonden` and `Edel og Wilhelm Daubenmerkls Almenvelgørende Fond´.
All authors have declared no conflict of interest.
Format based on the Ottawa Personal Decision Guide © 2000, A O'Connor, D Stacey, University of Ottawa, CA.
For more information contact Marie Louise Thise Rasmussen email: marie.louise.thise.rasmussen.01@regionh.dk
Benefits and harms data and data on patient preferences to value clarification:
DEFACTUM. Outpatient Parenteral Antibiotic Therapy – A Health Technology Assessment. Aarhus: DEFACTUM, 2021;
Staples, JA; et all. Outpatient versus inpatient intravenous antimicrobial therapy: a population-based observational cohort study of adverse events and costs. Clin Infect Dis. 2022
Patient preferences are also inspired by other decision aids assessing the location of treatment and developed with the Ottawa Decision Support Framework.
