# Supplementary material for: Deciding on the location for receiving parenteral antimicrobial therapy: development and preliminary testing of a patient decision aid
Source: BMC Health Serv Res. 2025 Sep 30;25:1240. doi: 10.1186/s12913-025-13434-w (PMC12482543; doi:10.1186/s12913-025-13434-w)
Supplement: Supplementary file 4 — Supplementary Material 4 [file 12913_2025_13434_MOESM4_ESM.pdf]

Region Hovedstaden

Center for Regional Udvikling

De Videnskabsetiske Komiteer
for Region Hovedstaden
Regionsgården
Kongens Vænge 2
3400 Hillerød

Opgang B + D
Telefon 38666395
Direkte 38666320
Mail vek@regionh.dk

Journal-nr.: 20068796

Dato: 16-10-2020

Ph.d.studerende Marie Louise Thise Rasmussen

Akutmodtagelsen

Herlev og Gentofte Hospital

marie.louise.thise.rasmussen.01@regionh.dk

Let the patient be heard

Du har ved mail af 14. oktober 2020 spurgt, om ovennævnte projekt skal anmeldes til det videnskabsetiske komitesystem.

I vil undersøge, hvordan patienter medinddrages omkring deres pleje og behandling i akutafdelinger, og I vil udvikle og afprøve et redskab til bedre og mere systematisk involvering af patienterne. Metoderne er spørgeskema- og interviewundersøgelser samt dialog mellem patient og personale baseret på en nyudviklet dialog-model.

Jeg har vurderet, at der ikke er tale om et sundhedsvidenskabeligt forskningsprojekt som dette er defineret i komitélovens § 2[1].

Projektet er derfor ikke anmeldelsespligtigt, jf. komitélovens § 1, stk. 4 og kan iværksættes uden tilladelse fra De Videnskabsetiske Komiteer for Region Hovedstaden.

I Danmark har det videnskabsetiske komitesystem til opgave at vurdere sundhedsvidenskabelige og sundhedsdatavidenskabelige forskningsprojekter.

Ved sundhedsvidenskabelige forskningsprojekter forstås projekter, der indebærer forsøg på levendefødte menneskelige individer, menneskelige kønsceller, der agtes anvendt til befrugtning, menneskelige befrugtede æg, fosteranlæg og fostre, væv, celler og arvebestanddele fra mennesker, fostre og lign. eller afdøde. Herunder omfattes kliniske forsøg med lægemidler på mennesker og klinisk afprøvning af medicinsk udstyr. Ved sundhedsdatavidenskabelige forskningsprojekter forstås forskning vedrørende særlige komplekse områder i afledte sensitive bioinformatiske data frembragt ved omfattende kortlægning af arvemassen eller billeddiagnostik i forbindelse med forsøg eller klinisk diagnostik af patienter.

Sundhedsvidenskabelig forskning omhandler primært forskning inden for de lægevidenskabelige fag, den kliniske og den socialmedicinsk-epidemiologiske forskning. Begrebet omfat-

---

[1] Afgørelsen er truffet efter lov lovbekendtgørelse nr. 1083 af 15/09/2017 med senere ændringer

ter, udover forskning af de somatiske sygdomme, tillige de psykiatriske og de klinisk-psykologiske sygdomme og tilstandsformer. Herudover inddrages tilsvarende odontologisk og farmaceutisk forskning under begrebet.

Registerforskningsprojekter (bortset fra sundhedsdatavidenskabelige projekter), interviewundersøgelser og spørgeskemaundersøgelser skal kun anmeldes, hvis der indgår menneskeligt biologisk materiale i projektet.

Der ligger således ikke i afvisningen af at bedømme projektet nogen etisk stillingtagen eller negativ vurdering af dets indhold.

Behandling af personhenførbare oplysninger er omfattet af databeskyttelsesloven/persondataforordningen. Nærmere oplysning herom findes på Datatilsynets hjemmeside.

Klagevejledning:

Afgørelsen kan, jf. komitélovens § 26, stk. 1, indbringes for National Videnskabsetisk Komité, senest 30 dage efter afgørelsen er modtaget. National Videnskabsetisk Komité kan, af hensyn til sikring af forsøgspersonernes rettigheder, behandle elementer af projektet, som ikke er omfattet af selve klagen.

Klagen skal indbringes elektronisk og ved brug af digital signatur og kryptering, hvis protokollen indeholder fortrolige oplysninger. Dette kan ske på adressen: dketik@dketik.dk.

Klagen skal begrundes og være vedlagt kopi af Den Regionale Videnskabsetiske Komités afgørelse samt de sagsakter, som Den Regionale Videnskabsetiske Komité har truffet afgørelse på grundlag af.

NB: Der må ikke foretages ændringer i dokumenterne, som har været til behandling i komiteen, da sagen ellers vil blive sendt retur til komiteen.

Med venlig hilsen

Lone Gundelach

Chefkonsulent, cand.jur.

Lone.gundelach@regionh.dk
